# Radiation Necrosis Secondary to Trigeminal Nerve TomoTherapy: A Cautionary Case Study

**DOI:** 10.7759/cureus.243

**Published:** 2015-01-26

**Authors:** Andrew Montoure, Hasan Zaidi, John P Sheehy, Andrew G Shetter, Robert Spetzler

**Affiliations:** 1 Department of Neurosurgery, Barrow Neurological Institute

**Keywords:** tomotherapy, trigeminal neuralgia, radiation necrosis

## Abstract

New radiation delivery modalities have recently challenged Gamma Knife surgery as the historic gold standard in the treatment of trigeminal neuralgia (TN). TomoTherapy, a relative newcomer, has been approved by the U.S. FDA for various intracranial pathologies but is currently off label for the treatment of TN. A 73-year-old female presented with gait instability, intermittent headaches, and confusion. She was treated with TomoTherapy for refractory TN at an outside facility, which failed to reduce her symptoms. Magnetic resonance imaging demonstrated a lesion in the right mesial temporal lobe. A standard right anterior temporal lobectomy was performed and the final pathological report was notable for necrosis, gliosis, and edema consistent with a remote radiation injury. The patient improved postoperatively, but at her two-year follow up, she continued to have persistent bilateral TN and new onset seizures. Imaging revealed no new mass in the resection field. Stereotactic radiosurgery (SRS) is an evolving field with broadening indications, which makes it ever more important for physicians to be aware of differences between various SRS modalities. This case report highlights a cautionary example, and emphasizes the need for a more systematic evaluation of novel SRS methods before clinical application.

## Introduction

Trigeminal neuralgia (TN) is characterized by debilitating attacks of stabbing facial pain along the distribution of one or more branches of the trigeminal nerve [1-2]. Gamma Knife surgery is historically the most popular and well-characterized form of stereotactic radiosurgery (SRS) for TN, with several series indicating excellent clinical efficacy with acceptable morbidity. New radiosurgery delivery devices introduced over the last 20 years are challenging this approach, but as these methods gain traction, little guidance is available in the neurosurgical literature on their long-term adverse effects. Helical TomoTherapy (Accuray, Sunnyvale, CA) is one such novel form of SRS, which was introduced in 1994 and originally designed for intracranial lesions [3]. The patient is placed on a couch, which moves through a gantry containing a rotating LINAC system and is capable of intensity-modulated radiotherapy, that is placed at 90-degree angles with the computed tomography (CT) source and detector. An integrated CT can provide immediate pretreatment confirmation of target and radiation beam volume (stereotactic localization), while producing the treatment-planning image [4].

The U.S. Food and Drug Administration has approved TomoTherapy for intracranial tumors and certain vascular malformations, but its use is currently off-label in the treatment of patients with TN. Literature on TomoTherapy in the management of TN is sparse, although a single technical report describes suggested treatment parameters without clinical case examples [5]. Here, we present the first known report in the neurosurgical literature of a patient with TN who was treated with TomoTherapy at an outside facility, and who subsequently developed radiation necrosis of her adjacent mesial-temporal lobe.

## Case presentation

A 73-year-old female presented to Barrow Neurological Institute with complaints of gait instability, intermittent headaches, and confusion per the family. The patient denied any recent memory loss, weakness, visual changes, seizures, or other constitutional symptoms. Her past medical history was significant for medically refractory bilateral TN, Castleman’s disease status post-partial colectomy, and a thyroid nodule with hyperthyroidism. Multiple drug regimens for TN had failed to provide adequate symptom control, and the patient was currently on carbamazepine. One year before admission to our institution, she had undergone TomoTherapy for her more severe, right-sided TN symptoms. She was reportedly symptom-free in the first three months after treatment, but her attacks had since returned and become more intense than her pretreatment baseline.

Medical records from the outside hospital, including the operative report for the TomoTherapy procedure, were obtained with the patient’s consent. According to the report, non-gadolinium enhanced images obtained for treatment planning showed no evidence of an intracranial mass. The TomoTherapy machine was set at 6× photon energy mode, and a total of 4,000 cGy was delivered to the target site in two treatment sessions. We were unable to obtain information regarding the portion of the trigeminal nerve targeted, the volume of tissue that received the 4,000 cGy dose, or the isodose lines. At the five-week follow-up, the treating physician noted that the patient was pain-free.

**Figure 1:**
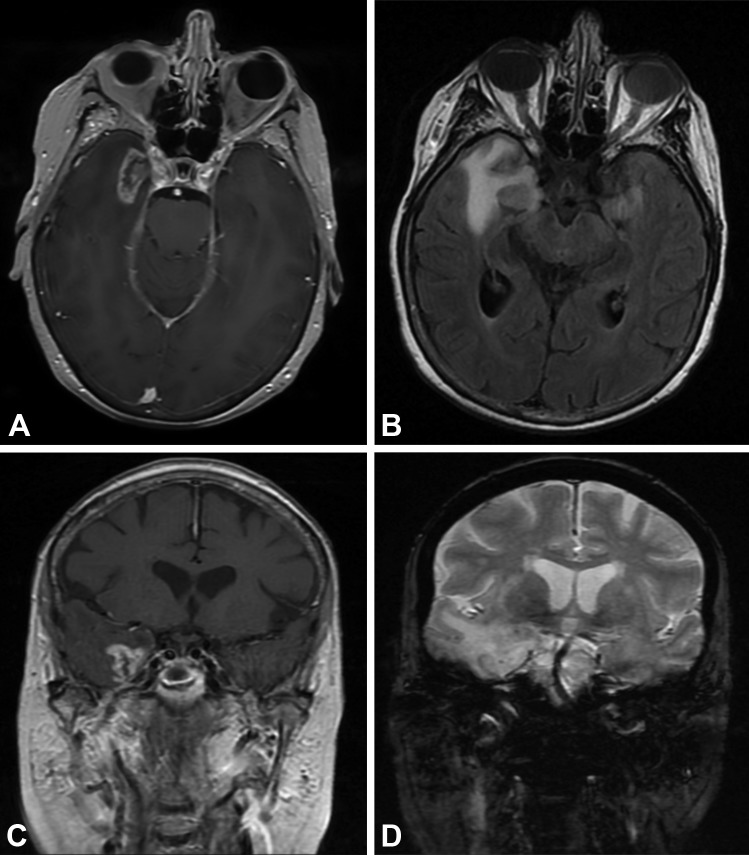
Radiation Necrosis Magnetic resonance images at presentation of a 73-year-old female with a history of trigeminal neuralgia, which was treated previously by TomoTherapy radiosurgery, shows significant mass effect in close proximity to Meckel’s cave. (A) Axial T1-weighted image with contrast; (B) axial FLAIR; (C) coronal T1-weighted image with contrast; and (D) coronal T2-weighted image. *Used with permission from Barrow Neurological Institute.*

Upon admission to our institution, the patient was confused and had evidence of a minimally diminished left upper-quadrant visual field. Hematological evaluation was notable for severe hyponatremia (124 mEq/L), which was slowly corrected over the course of 48 hours. During this time, a magnetic resonance image (MRI) study was ordered, and a 2.7x1.5x1.7 cm heterogeneously enhancing mass in the right mesial temporal lobe with perilesional edema was incidentally found (Figure 1).

Given her age and medical history, our differential diagnoses included metastatic disease, glioblastoma multiforme, or radiation necrosis. Informed patient consent was obtained after discussing the risks and benefits of observation versus open resection/biopsy. The patient elected to undergo surgical intervention. Given the patients’ age and imaging findings, our clinical suspicion for primary high-grade glioma was high. In these cases, the senior author (RFS) is aggressive in removal of tissue beyond margins for presumed micrometastatic disease if the lesion is located in non-eloquent territory. We therefore performed a standard right anterior temporal lobectomy, and several specimens were sent for pathological evaluation. Intraoperative interpretation of the specimen revealed necrotic and inflamed tissue. The frozen section demonstrated gray matter with edema, gliosis, and a large area of coagulation necrosis, vessels suggested fibrinoid degeneration, and no neoplasm was found. The formalin preparation showed a pattern of injury typical of that associated with radiation therapy injury. The final pathological analysis concluded necrosis, gliosis, and edema consistent with remote radiation injury.

**Figure 2:**
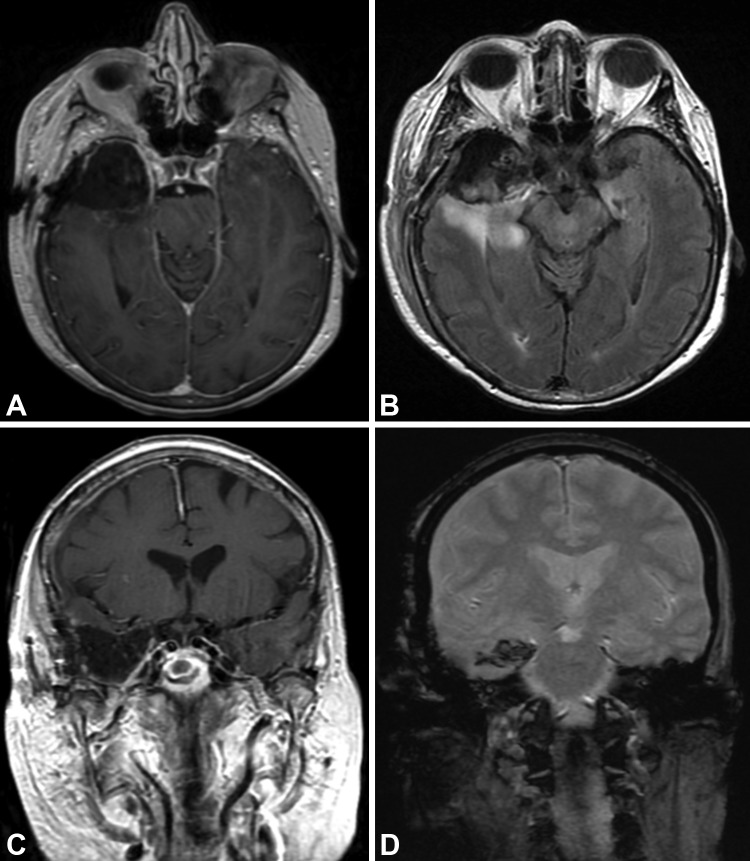
Postoperative Resection Postoperative magnetic resonance image showing excellent resection of enhancing portion, pathologically confirmed to be radiation necrosis. (A) Axial T1-weighted image with contrast; (B) axial FLAIR; (C) coronal T1-weighted image with contrast; and (D) coronal T2-weighted image.* Used with permission from Barrow Neurological Institute.*

Postoperatively, the patient had improvement in her confusion and remained neurologically intact at her two-week follow-up. The postoperative MRI showed expected postoperative changes (Figure 2).

At her two-year follow-up, the patient noted persistent bilateral TN symptoms as well as new onset of seizure activity necessitating an increased dose of antiepileptic medication, but without new lesions within her resection cavity.

## Discussion

We present here the first known report in the neurosurgical literature of sequelae of using TomoTherapy to treat a patient with medically refractory TN. This case should not only serve as a cautionary tale, but also emphasize that this relatively novel radiation delivery method requires a more rigorous evaluation before it is applied clinically to treat trigeminal neuralgia.

TomoTherapy is a popular tool for total marrow and lymphoid irradiation, multiple brain metastasis, and stereotactic body radiotherapy [6]. Advantages of TomoTherapy include increased mechanical degree of freedom, the ability to deliver highly conformal doses to multiple targets simultaneously, decreased radiation toxicity to the scalp and middle ear, improved coverage to the base of skull and craniospinal lesions, and volumetric source imaging for both localization and treatment planning [4]. One of the advantages of other SRS methods is the ability to produce noncoplanar beams, which allows for improved dose distribution. TomoTherapy does not have this capability, and therefore conventional SRS is the preferred method for small intracranial lesions [6]. Several adverse events have been described after TomoTherapy for intracranial lesions, but no large clinical series are available in the literature directly comparing TomoTherapy with more traditional methods of SRS [7].

The objective of SRS is to deliver a highly concentrated dose of radiation to the target while avoiding exposure to surrounding healthy tissue—this is referred to as dose gradient or cutoff. Having a steep dose fall-off is critical, especially when dealing with small target structures, such as the trigeminal nerve. TomoTherapy has limited resolution due to the thickness of the slices after each rotation; this can then lead to injury of surrounding neurovascular structures due to an imprecise dose gradient [6]. When compared with other SRS methods, TomoTherapy results in higher radiation exposure to normal tissue surrounding the target. Gamma Knife surgery (GKS) delivers 192 or 201 simultaneous beams from varying directions, allowing the noncoplanar arcs to achieve a rather concise and isotropic dose gradient, sparing tissue outside the targeted zone [6]. Penagaricano, et al. compared the conformity index and volume of normal tissue receiving radiation between TomoTherapy and GKS. GKS was found superior in both categories, having an optimal conformity index and a smaller volume of irradiated normal tissue (eye, optic nerve, optic chiasm) [8]. CyberKnife (Accuray, Sunnyvale, California, U.S.A.) also showed similar dose cutoff gradients to GKS, and had significantly better normal tissue sparing when compared with TomoTherapy [9].

## Conclusions

To our knowledge, this is the first reported case of radiation necrosis secondary to TomoTherapy for trigeminal neuralgia. Stereotactic radiosurgery is an evolving field within both neurological surgery and radiation oncology. Indications are broadening, which makes it ever more important for physicians to be aware of differences between SRS modalities. This case report highlights a cautionary example, and emphasizes the need for a more systematic evaluation of novel SRS methods before clinical application.
